# Author Correction: Multi-ancestry genome-wide association analyses improve resolution of genes and pathways influencing lung function and chronic obstructive pulmonary disease risk

**DOI:** 10.1038/s41588-023-01531-7

**Published:** 2023-09-25

**Authors:** Nick Shrine, Abril G. Izquierdo, Jing Chen, Richard Packer, Robert J. Hall, Anna L. Guyatt, Chiara Batini, Rebecca J. Thompson, Chandan Pavuluri, Vidhi Malik, Brian D. Hobbs, Matthew Moll, Wonji Kim, Ruth Tal-Singer, Per Bakke, Katherine A. Fawcett, Catherine John, Kayesha Coley, Noemi Nicole Piga, Alfred Pozarickij, Kuang Lin, Iona Y. Millwood, Zhengming Chen, Liming Li, China Kadoorie Biobank Collaborative Group, Alfred Pozarickij, Kuang Lin, Iona Y. Millwood, Zhengming Chen, Liming Li, Robin G. Walters, Sara R. A. Wijnant, Lies Lahousse, Guy Brusselle, Andre G. Uitterlinden, Ani Manichaikul, Elizabeth C. Oelsner, Stephen S. Rich, R. Graham Barr, Shona M. Kerr, Veronique Vitart, Michael R. Brown, Matthias Wielscher, Medea Imboden, Ayoung Jeong, Traci M. Bartz, Sina A. Gharib, Claudia Flexeder, Stefan Karrasch, Christian Gieger, Annette Peters, Beate Stubbe, Xiaowei Hu, Victor E. Ortega, Deborah A. Meyers, Eugene R. Bleecker, Stacey B. Gabriel, Namrata Gupta, Albert Vernon Smith, Jian’an Luan, Jing-Hua Zhao, Ailin F. Hansen, Arnulf Langhammer, Cristen Willer, Laxmi Bhatta, David Porteous, Blair H. Smith, Archie Campbell, Tamar Sofer, Jiwon Lee, Martha L. Daviglus, Bing Yu, Elise Lim, Hanfei Xu, George T. O’Connor, Gaurav Thareja, Omar M. E. Albagha, The Qatar Genome Program Research (QGPR) Consortium, Said I. Ismail, Wadha Al-Muftah, Radja Badji, Hamdi Mbarek, Dima Darwish, Tasnim Fadl, Heba Yasin, Maryem Ennaifar, Rania Abdellatif, Fatima Alkuwari, Muhammad Alvi, Yasser Al-Sarraj, Chadi Saad, Asmaa Althani, Karsten Suhre, Raquel Granell, Tariq O. Faquih, Pieter S. Hiemstra, Annelies M. Slats, Benjamin H. Mullin, Jennie Hui, Alan James, John Beilby, Karina Patasova, Pirro Hysi, Jukka T. Koskela, Annah B. Wyss, Jianping Jin, Sinjini Sikdar, Mikyeong Lee, Sebastian May-Wilson, Nicola Pirastu, Katherine A. Kentistou, Peter K. Joshi, Paul R. H. J. Timmers, Alexander T. Williams, Robert C. Free, Xueyang Wang, John L. Morrison, Frank D. Gilliland, Zhanghua Chen, Carol A. Wang, Rachel E. Foong, Sarah E. Harris, Adele Taylor, Paul Redmond, James P. Cook, Anubha Mahajan, Lars Lind, Teemu Palviainen, Terho Lehtimäki, Olli T. Raitakari, Jaakko Kaprio, Taina Rantanen, Kirsi H. Pietiläinen, Simon R. Cox, Craig E. Pennell, Graham L. Hall, W. James Gauderman, Chris Brightling, James F. Wilson, Tuula Vasankari, Tarja Laitinen, Veikko Salomaa, Dennis O. Mook-Kanamori, Nicholas J. Timpson, Eleftheria Zeggini, Josée Dupuis, Caroline Hayward, Ben Brumpton, Claudia Langenberg, Stefan Weiss, Georg Homuth, Carsten Oliver Schmidt, Nicole Probst-Hensch, Marjo-Riitta Jarvelin, Alanna C. Morrison, Ozren Polasek, Igor Rudan, Joo-Hyeon Lee, Ian Sayers, Emma L. Rawlins, Frank Dudbridge, Edwin K. Silverman, David P. Strachan, Robin G. Walters, Andrew P. Morris, Stephanie J. London, Michael H. Cho, Louise V. Wain, Ian P. Hall, Martin D. Tobin

**Affiliations:** 1 Department of Population Health Sciences, University of Leicester, Leicester, UK; 2 Division of Respiratory Medicine and NIHR Nottingham Biomedical Research Centre, University of Nottingham, Nottingham, UK; 3 Leicester National Institute for Health and Care Research, Biomedical Research Centre, Glenfield Hospital, Leicester, UK; 4 Channing Division of Network Medicine, Division of Pulmonary and Critical Care Medicine, Department of Medicine, Brigham and Women’s Hospital, Boston, MA USA; 5 Harvard Medical School, Boston, MA USA; 6 COPD Foundation, Washington DC, USA; 7 Department of Clinical Science, Unversity of Bergen, Bergen, Norway; 8 Nuffield Department of Population Health, University of Oxford, Oxford, UK; 9 MRC Population Health Research Unit, University of Oxford, Oxford, UK; 10 Department of Epidemiology and Biostatistics, School of Public Health, Peking University Health Science Center, Beijing, China; 11 Department of Respiratory Diseases, Ghent Universital Hospital, Ghent, Belgium; 12 Department of Bioanalysis, Faculty of Pharmaceutical Sciences, Ghent University, Ghent, Belgium; 13 Department of Epidemiology, Eramus Medical Center, Rotterdam, The Netherlands; 14 Department of Internal Medicine, Eramus Medical Center, Rotterdam, The Netherlands; 15 Center for Public Health Genomics, University of Virginia, Charlottesville, VA USA; 16 Department of Medicine, Columbia University Medical Center, New York, NY USA; 17 Medical Research Council Human Genetics Unit, Institute of Genetics and Cancer, University of Edinburgh, Edinburgh, UK; 18 Human Genetics Center, Department of Epidemiology, Human Genetics, and Environmental Sciences, School of Public Health, The University of Texas Health Science Center at Houston, Houston, TX USA; 19 MRC Centre for Environment and Health, Department of Epidemiology and Biostatistics, School of Public Health, Imperial College London, London, UK; 20 Department of Epidemiology and Public Health, Swiss Tropical and Public Health Institute, Allschwil, Switzerland; 21 Department of Public Health, University of Basel, Basel, Switzerland; 22 Cardiovascular Health Research Unit, Departments of Medicine and Biostatistics, University of Washington, Seattle, WA USA; 23 Computational Medicine Core, Center for Lung Biology, Division of Pulmonary, Critical Care and Sleep Medicine, Department of Medicine, University of Washington, Seattle, WA USA; 24 Institute and Clinic for Occupational, Social and Environmental Medicine, University Hospital, LMU Munich, Munich, Germany; 25 Comprehensive Pneumology Center Munich (CPC-M), German Center for Lung Research (DZL), Munich, Germany; 26 Institute of Epidemiology, Helmholtz Zentrum München–German Research Center for Environmental Health, Neuherberg, Germany; 27 Research Unit of Molecular Epidemiology, Helmholtz Zentrum München–German Research Center for Environmental Health, Neuherberg, Germany; 28 Institute for Medical Information Processing, Biometry and Epidemiology, Medical Faculty, Ludwig Maximilian University, Munich, Germany; 29 Department of Internal Medicine B–Cardiology, Intensive Care, Pulmonary Medicine and Infectious Diseases, University Medicine Greifswald, Greifswald, Germany; 30 Division of Respiratory Medicine, Department of Internal Medicine, Center for Individualized Medicine, Mayo Clinic, Scottsdale, AZ USA; 31 Department of Medicine, University of Arizona, Tucson, AZ USA; 32 Broad Institute of MIT and Harvard, Cambridge, MA USA; 33 Department of Biostatistics, University of Michigan School of Public Health, Ann Arbor, MI USA; 34 Center for Statistical Genetics, University of Michigan School of Public Health, Ann Arbor, MI USA; 35 MRC Epidemiology Unit, Institute of Metabolic Science, School of Clinical Medicine, University of Cambridge, Cambridge, UK; 36 Department of Public and Primary Care, Heart and Lung Research Institute, University of Cambridge, Cambridge, UK; 37 K.G. Jebsen Center for Genetic Epidemiology, Department of Public Health and Nursing, NTNU Norwegian University of Science and Technology, Trondheim, Norway; 38 HUNT Research Centre, Department of Public Health and Nursing, NTNU Norwegian University of Science and Technology, Levanger, Norway; 39 Levanger Hospital, Nord-Trøndelag Hospital Trust, Levanger, Norway; 40 Division of Cardiology, Department of Internal Medicine, University of Michigan, Ann Arbor, MI USA; 41 Department of Biostatistics and Center for Statistical Genetics, University of Michigan, Ann Arbor, MI USA; 42 Department of Human Genetics, University of Michigan, Ann Arbor, MI USA; 43 Centre for Genomic and Experimental Medicine, Institute of Genetics and Cancer, University of Edinburgh, Edinburgh, UK; 44 Division of Population Health and Genomics, Ninewells Hospital and Medical School, University of Dundee, Dundee, UK; 45 Division of Sleep and Circadian Disorders, Brigham and Women’s Hospital, Boston, MA USA; 46 Department of Medicine, Harvard Medical School, Boston, MA USA; 47 Department of Biostatistics, Harvard T.H. Chan School of Public Health, Boston, MA USA; 48 Institute for Minority Health Research, University of Illinois at Chicago, Chicago, IL USA; 49 Human Genetics Center, Department of Epidemiology, Human Genetics and Environmental Sciences, School of Public Health, University of Texas Health Science Center at Houston, Houston, TX USA; 50 Department of Biostatistics, School of Public Health, Boston University, Boston, MA USA; 51 Pulmonary Center, School of Medicine, Boston University, Boston, MA USA; 52 Bioinformatics Core, Weill Cornell Medicine–Qatar, Education City, Doha, Qatar; 53 College of Health and Life Sciences, Hamad Bin Khalifa University, Doha, Qatar; 54 Center for Genomic and Experimental Medicine, Institute of Genetics and Cancer, University of Edinburgh, Edinburgh, UK; 55 Wellcome Sanger Institute, Cambridge, UK; 56 Department of Biophysics and Physiology, Weill Cornell Medicine, New York, NY USA; 57 MRC Integrative Epidemiology Unit (IEU), Population Health Sciences, Bristol Medical School, University of Bristol, Bristol, UK; 58 Department of Clinical Epidemiology, Leiden University Medical Center, Leiden, The Netherlands; 59 Department of Pulmonology, Leiden University Medical Center, Leiden, The Netherlands; 60 Department of Endocrinology and Diabetes, Sir Charles Gairdner Hospital, Nedlands, Western Australia Australia; 61 School of Biomedical Sciences, University of Western Australia, Crawley, Western Australia Australia; 62 Busselton Population Medical Research Institute, QEII Medical Centre, Nedlands, Western Australia Australia; 63 School of Population and Global Health, University of Western Australia, Crawley, Western Australia Australia; 64 PathWest Laboratory Medicine of WA, Nedlands, Western Australia Australia; 65 Department of Twin Research and Genetic Epidemiology, King’s College London School of Medicine, London, UK; 66 Division of Respiratory Medicine, Department of Medicine Solna, Karolinska Institutet, Karolinska University Hospital, Stockholm, Sweden; 67 UCL Institute of Ophthalmology, University College London, London, UK; 68 Institute for Molecular Medicine Finland (FIMM), University of Helsinki, Helsinki, Finland; 69 Epidemiology Branch, National Institute of Environmental Health Sciences, National Institutes of Health, Department of Health and Human Services, Research Triangle Park, NC USA; 70 Westat, Durham, NC USA; 71 Department of Mathematics and Statistics, Old Dominion University, Norfolk, VA USA; 72 Centre for Global Health Research, Usher Institute for Population Health Sciences and Informatics, University of Edinburgh, Edinburgh, UK; 73 Centre for Cardiovascular Sciences, Queen’s Medical Research Institute, University of Edinburgh, Edinburgh, UK; 74 Department of Respiratory Sciences, University of Leicester, Leicester, UK; 75 Department of Population and Public Health Sciences, Keck School of Medicine, University of Southern California, Los Angeles, CA USA; 76 School of Medicine and Public Health, College of Health, Medicine and Wellbeing, University of Newcastle, Newcastle, New South Wales Australia; 77 Hunter Medical Research Institute, Newcastle, New South Wales Australia; 78 Wal-yan Respiratory Research Centre, Telethon Kids Institute, Perth, Western Australia Australia; 79 School of Allied Health, Faculty of Health Sciences, Curtin University, Perth, Western Australia Australia; 80 Lothian Birth Cohorts group, Department of Psychology, University of Edinburgh, Edinburgh, UK; 81 Department of Health Data Science, University of Liverpool, Liverpool, UK; 82 Wellcome Centre for Human Genetics, University of Oxford, Oxford, UK; 83 Genentech, South San Francisco, CA USA; 84 Department of Medical Sciences, Uppsala University, Uppsala, Sweden; 85 Institute for Molecular Medicine Finland–FIMM, University of Helsinki, Helsinki, Finland; 86 Department of Clinical Chemistry, Fimlab Laboratories, and Finnish Cardiovascular Research Center–Tampere, Faculty of Medicine and Health Technology, Tampere University, Tampere, Finland; 87 Department of Clinical Physiology and Nuclear Medicine, Turku University Hospital, Turku, Finland; 88 Research Centre of Applied and Preventive Cardiovascular Medicine, University of Turku, Turku, Finland; 89 Faculty of Sport and Health Sciences, University of Jyvaskyla, Jyvaskyla, Finland; 90 Obesity Research Unit, Research Program for Clinical and Molecular Metabolism, Faculty of Medicine, University of Helsinki, Helsinki, Finland; 91 Obesity and Abdominal Centers, Helsinki University Hospital and University of Helsinki, Helsinki, Finland; 92 Department of Maternity and Gynaecology, John Hunter Hospital, Newcastle, New South Wales Australia; 93 Department of Infection, Inflammation and Immunity, Institute for Lung Health, University of Leicester, Leicester, UK; 94 MRC Human Genetics Unit, Institute of Genetics and Cancer, University of Edinburgh, Western General Hospital, Edinburgh, UK; 95 FILHA–Finnish Lung Health Association, Helsinki, Finland; 96 Department of Respiratory Diseases and Allergology, University of Turku, Turku, Finland; 97 Administration Center, Tampere University Hospital and University of Tampere, Tampere, Finland; 98 Department of Public Health and Welfare, Finnish Institute for Health and Welfare, Helsinki, Finland; 99 Department of Public Health and Primary Care, Leiden University Medical Center, Leiden, The Netherlands; 100 ALSPAC, Department of Population Health Sciences, Bristol Medical School, University of Bristol, Bristol, UK; 101 Institute of Translational Genomics, Helmholtz Zentrum München–German Research Center for Environmental Health, Neuherberg, Germany; 102 Technical University of Munich (TUM) and Klinikum Rechts der Isar, TUM School of Medicine, Munich, Germany; 103 Department of Epidemiology, Biostatistics, and Occupational Health, School of Population and Global Health, McGill University, Montreal, Quebec Canada; 104 Clinic of Medicine, St. Olavs Hospital, Trondheim University Hospital, Trondheim, Norway; 105 Precision Healthcare University Research Institute, Queen Mary University of London, London, UK; 106 Computational Medicine, Berlin Institute of Health at Charité, Universitätsmedizin Berlin, Berlin, Germany; 107 Interfaculty Institute for Genetics and Functional Genomics, Department of Functional Genomics, University Medicine Greifswald, Greifswald, Germany; 108 Institute for Community Medicine, SHIP–Clinical Epidemiological Research, University Medicine Greifswald, Greifswald, Germany; 109 Center for Life Course Health Research, Faculty of Medicine, University of Oulu, Oulu, Finland; 110 Biocenter Oulu, University of Oulu, Oulu, Finland; 111 Unit of Primary Health Care, Oulu University Hospital, OYS, Oulu, Finland; 112 School of Medicine, University of Split, Split, Croatia; 113 Centre for Global Health, Usher Institute, University of Edinburgh, Edinburgh, UK; 114 Jeffrey Cheah Biomedical Centre, Wellcome–MRC Cambridge Stem Cell Institute, University of Cambridge, Cambridge, UK; 115 Department of Physiology, Development and Neuroscience, University of Cambridge, Cambridge, UK; 116 Wellcome Trust–CRUK Gurdon Institute and Department of Physiology, Development and Neuroscience, University of Cambridge, Cambridge, UK; 117 Population Health Research Institute, St George’s University of London, London, UK; 118 Centre for Genetics and Genomics Versus Arthritis, Division of Musculoskeletal and Dermatological Sciences, Centre for Musculoskeletal Research, The University of Manchester, Manchester, UK; 119 Qatar Genome Program, Qatar Foundation Research Development and Innovation, Qatar Foundation, Doha, Qatar; 120 Qatar Biobank for Medical Research, Qatar Foundation, Doha, Qatar; 121 Integrated Genomics Services, Sidra Medicine, Doha, Qatar; 122 Applied Bioinformatics Core, Sidra Medicine, Doha, Qatar; 123 Biomedical Informatics, Sidra Medicine, Doha, Qatar; 124 Microbiome and Biomarkers Discovery Laboratory, Sidra Medicine, Doha, Qatar; 125 College of Health Sciences, Qatar University, Doha, Qatar; 126 Molecular Genetics Laboratory, Hamad Medical Corporation, Doha, Qatar; 127 Department of Genetic Medicine, Microbiology and Immunology, Weill Cornell Medicine–Qatar, Doha, Qatar; 128 Research Branch, Sidra Medicine, Doha, Qatar; 129 Department of Human Genetics, Sidra Medicine, Doha, Qatar; 130 Weill Cornell Medicine–Qatar, Doha, Qatar; 131 Genomic Medicine Laboratory, Sidra Medicine, Doha, Qatar; 132 Medical and Population Genomics Laboratory, Sidra Medicine, Doha, Qatar; 133 Clinical Research Centre, Sidra Medicine, Doha, Qatar

**Keywords:** Genome-wide association studies, Respiratory tract diseases

Correction to: *Nature Genetics* 10.1038/s41588-023-01314-0, published online 13 March 2023.

In the version of the article initially published, the sample sizes in the main text and Supplementary Tables 1 and 2 were incorrect. In the abstract, the last paragraph of the Introduction, the first paragraph of the Results, the top box in Figure 1a and the Supplementary Information, the total sample size has been corrected from 580,869 to 588,452 participants and the size of the European cohort from 468,062 to 475,645. Some of the effect sizes in Supplementary Table 14 (columns W, Z, AC, AF) had the wrong sign. There was also an error in Supplementary Table 3 where the sample size instead of the variant count was shown for EXCEED. The errors do not affect the conclusions of the study. Additionally, two acknowledgments for use of INTERVAL pQTL and Lung eQTL consortium data were omitted from the Supplementary Information. These errors have been corrected in the Supplementary Information and HTML and PDF versions of the article.

