# Supplementary material for: Multi-ancestry genome-wide association analyses improve resolution of genes and pathways influencing lung function and chronic obstructive pulmonary disease risk
Source: Nat Genet. 2023 Mar 13;55(3):410–22. doi: 10.1038/s41588-023-01314-0 (PMC10011137; doi:10.1038/s41588-023-01314-0)
Supplement: Supplementary file 2 — Reporting Summary [file 41588_2023_1314_MOESM2_ESM.pdf]

nature portfolio

Corresponding author(s): Nick Shrine

Last updated by author(s): Dec 22, 2022

Reporting Summary

Nature Portfolio wishes to improve the reproducibility of the work that we publish. This form provides structure for consistency and transparency in reporting. For further information on Nature Portfolio policies, see our Editorial Policies and the Editorial Policy Checklist.

Statistics

For all statistical analyses, confirm that the following items are present in the figure legend, table legend, main text, or Methods section.

n/a	Confirmed	
☐	☒	The exact sample size (*n*) for each experimental group/condition, given as a discrete number and unit of measurement
☐	☒	A statement on whether measurements were taken from distinct samples or whether the same sample was measured repeatedly
☐	☒	The statistical test(s) used AND whether they are one- or two-sided<br>*Only common tests should be described solely by name; describe more complex techniques in the Methods section.*
☐	☒	A description of all covariates tested
☐	☒	A description of any assumptions or corrections, such as tests of normality and adjustment for multiple comparisons
☐	☒	A full description of the statistical parameters including central tendency (e.g. means) or other basic estimates (e.g. regression coefficient) AND variation (e.g. standard deviation) or associated estimates of uncertainty (e.g. confidence intervals)
☐	☒	For null hypothesis testing, the test statistic (e.g. *F*, *t*, *r*) with confidence intervals, effect sizes, degrees of freedom and *P* value noted<br>*Give P values as exact values whenever suitable.*
☐	☒	For Bayesian analysis, information on the choice of priors and Markov chain Monte Carlo settings
☒	☐	For hierarchical and complex designs, identification of the appropriate level for tests and full reporting of outcomes
☐	☒	Estimates of effect sizes (e.g. Cohen's *d*, Pearson's *r*), indicating how they were calculated

Our web collection on statistics for biologists contains articles on many of the points above.

Software and code

Policy information about availability of computer code

Data collection	None
Data analysis	BOLT-LMM v2.3.4, R v4.1.0, ADMIXTURE v1.3.0, METAL v2018-08-28, LDSC v1.0.0, GCTA v1.93.2beta, PLINK v1.9, PLINK v2.0, ANNOVAR v2014-11-12, MR-MEGA v0.2, GARFIELD v2, fGWAS v0.3.6, coloc v3.2-1

For manuscripts utilizing custom algorithms or software that are central to the research but not yet described in published literature, software must be made available to editors and reviewers. We strongly encourage code deposition in a community repository (e.g. GitHub). See the Nature Portfolio guidelines for submitting code & software for further information.

Data

Policy information about availability of data

All manuscripts must include a data availability statement. This statement should provide the following information, where applicable:

- Accession codes, unique identifiers, or web links for publicly available datasets
- A description of any restrictions on data availability
- For clinical datasets or third party data, please ensure that the statement adheres to our policy

Genome-wide summary statistics for the multi-ancestry meta-analysis are available at GWAS catalog (https://www.ebi.ac.uk/gwas/); accession codes GCST90244092, GCST90244093, GCST90244094, GCST90244095

Human research participants

Policy information about studies involving human research participants and Sex and Gender in Research.

Reporting on sex and gender	Self-reported sex was used as a covariate in the GWAS in each of the 49 contributing cohorts. In total there were 256,850 males and 324,055 females included in this study. Individual level sex information is not available.
Population characteristics	Genotypic information, spirometry, age, sex, height and ancestry principal components were used in the 49 contributing cohorts and questionnaire results and primary and secondary care diagnoses were used for the PheWAS in UK Biobank. Covariates used in association testing were age (at spirometry measurement), age2, sex, and height, and also axes of genetic ancestry to model heterogeneity due to ancestry. Covariate-relevant population characteristics are summarised in Supplementary Table 2.
Recruitment	Recruitment of participants in the 49 cohorts is described in the Supplementary Note.
Ethics oversight	The organisations approving the study protocol in each of the cohorts is given in the Supplementary Note.

Note that full information on the approval of the study protocol must also be provided in the manuscript.

Field-specific reporting

Please select the one below that is the best fit for your research. If you are not sure, read the appropriate sections before making your selection.

☒ Life sciences ☐ Behavioural & social sciences ☐ Ecological, evolutionary & environmental sciences

For a reference copy of the document with all sections, see nature.com/documents/nr-reporting-summary-flat.pdf

Life sciences study design

All studies must disclose on these points even when the disclosure is negative.

Sample size	In order to maximise power, we used all samples available in 49 contributing cohorts with good measures of lung function and that had been genotyped giving a total sample size of 580,869
Data exclusions	Cohorts excluded samples with low-quality lung function measurements or samples that failed genotyping quality control.
Replication	To maximise power, all cohorts were used in discovery and we assessed the heterogeneity of effect estimates across contributing cohorts, and the extent to which heterogeneity was attributable to ancestry. After accounting for heterogeneity due to ancestry 93 of 1020 signals reported showed residual heterogeneity of effects across cohorts.
Randomization	This was an observational study; due to random allocation of genetic variants during gamete production genetic association studies of germline association are not expected to be subject to the confounding and reverse causation typically seen in traditional observational epidemiology studies
Blinding	Blinding was not necessary as the analysts were not involved in the measurement of lung function or genotypes and all samples are anonymised.

Reporting for specific materials, systems and methods

We require information from authors about some types of materials, experimental systems and methods used in many studies. Here, indicate whether each material, system or method listed is relevant to your study. If you are not sure if a list item applies to your research, read the appropriate section before selecting a response.

Materials & experimental systems

n/a	Involved in the study	
☒	☐	Antibodies
☒	☐	Eukaryotic cell lines
☒	☐	Palaeontology and archaeology
☒	☐	Animals and other organisms
☒	☐	Clinical data
☒	☐	Dual use research of concern

Methods

n/a	Involved in the study	
☒	☐	ChIP-seq
☒	☐	Flow cytometry
☒	☐	MRI-based neuroimaging
